# Supplementary material for: Diversity in Cell Morphology, Composition, and Function among Adipose Depots in River Buffaloes
Source: Int J Mol Sci. 2023 May 7;24(9):8410. doi: 10.3390/ijms24098410 (PMC10179058; doi:10.3390/ijms24098410)
Supplement: Supplementary file 1 [file ijms-24-08410-s001.zip › Supplementary Materials.pdf]

International Journal of
Molecular Sciences

MDPI

Article

Diversity in Cell Morphology, Composition, and Function among Adipose Depots in River Buffaloes

Xintong Yang †, Ruirui Zhu †, Ziyi Song, Deshun Shi * and Jieping Huang *

Supplementary Materials

The following supporting information can be downloaded at: www.mdpi.com/xxx/s1,

Figure S1: Relationship among samples or between modules and traits in WGCNA. (a) Cluster analysis using transcripts with average FPKM ≥2 across the 36 AT samples. (b) Relationship be-tween samples and SSAT. (c) Relationship between samples and SAT. (d) Relationship between samples and UFA.

Figure S2: Regulatory network of candidate genes in corresponding category based on their weight in WGCNA. (a) Regulatory network of candidate genes in ECM associated items in yellow and greenyellow modules. (b) Regulatory network of candidate genes in mitochondrial inner membrane/matrix and ATP binding/enzymatic activity in brown module. (c) Regulatory network of candidate genes in bta01100:Metabolic pathways in brown module. (d) Regulatory network of candidate genes in GO:0005743~mitochondrial inner membrane in brown module. (e) Regulatory network of candidate genes in bta00564:Glycerophospholipid metabolism pathway in blue module. (f) Regulatory network of candidate genes in the nine categories included in FA metabolic in blue module. Genes with high expression (top 30) in corresponding AT depot are presented for each regulatory network.

Figure S3: Expressional profile of the thermogenesis and lipolysis associated genes based on RNA sequencing.

Table S1: Details of quantity control and mapping of RNA sequencing.

Table S2: Identification and expression of mRNA in all the 36 adipose tissues.

Table S3: Differentially expressed genes between subcutaneous and visceral adipose depots.

Table S4: Functional enrichment by the genes upregulated in subcutaneous adipose depot.

Table S5: Functional enrichment by the genes upregulated in visceral adipose depot.

Table S6: Hox paralogs identified in the six adipose depots.

Table S7: Functional enrichment by the genes involved in the yellow and green-yellow modules.

Table S8: Functional enrichment by the genes involved in the brown module.

Table S9: Functional enrichment by the genes involved in the blue module.

Table S10: Functional enrichment by the genes involved in the turquoise module.

Table S11: Functional enrichment by the genes involved in the pink and red modules.

Table S12: Genes in candidate items.

Table S13: Details of buffaloes used in this study.
