# Supplementary figures and images for: Diversity in Cell Morphology, Composition, and Function among Adipose Depots in River Buffaloes
Source: Int J Mol Sci. 2023 May 7;24(9):8410. doi: 10.3390/ijms24098410 (PMC10179058; doi:10.3390/ijms24098410)

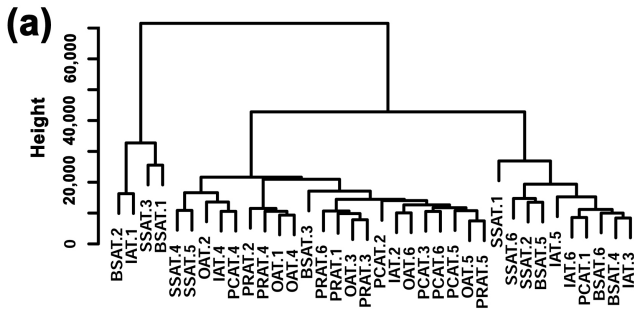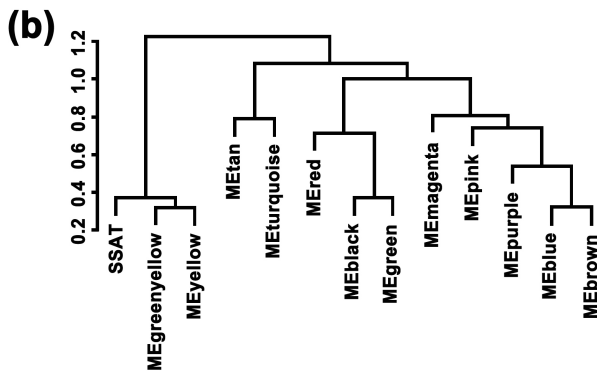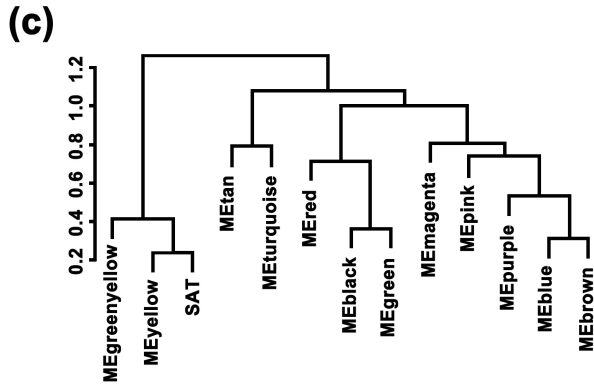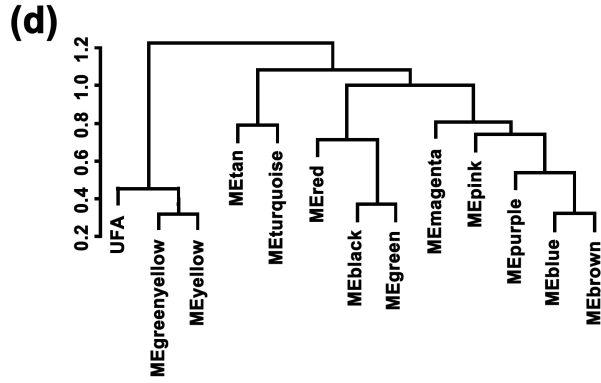

Supplement: Supplementary file 1 [file ijms-24-08410-s001.zip › Figure S1.pdf]

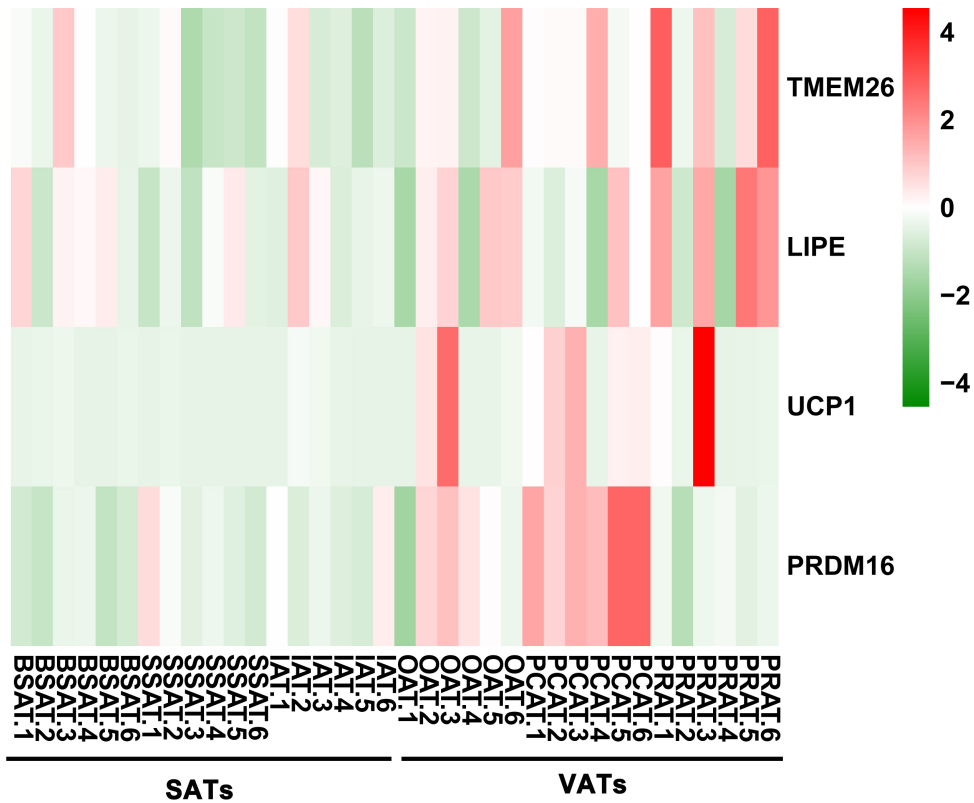

Supplement: Supplementary file 1 [file ijms-24-08410-s001.zip › Figure S3.pdf]
