# Supplementary material for: Diversity in Cell Morphology, Composition, and Function among Adipose Depots in River Buffaloes
Source: Int J Mol Sci. 2023 May 7;24(9):8410. doi: 10.3390/ijms24098410 (PMC10179058; doi:10.3390/ijms24098410)
Supplement: Supplementary file 1 [file Figure S2.pdf › Figure S2.pdf]

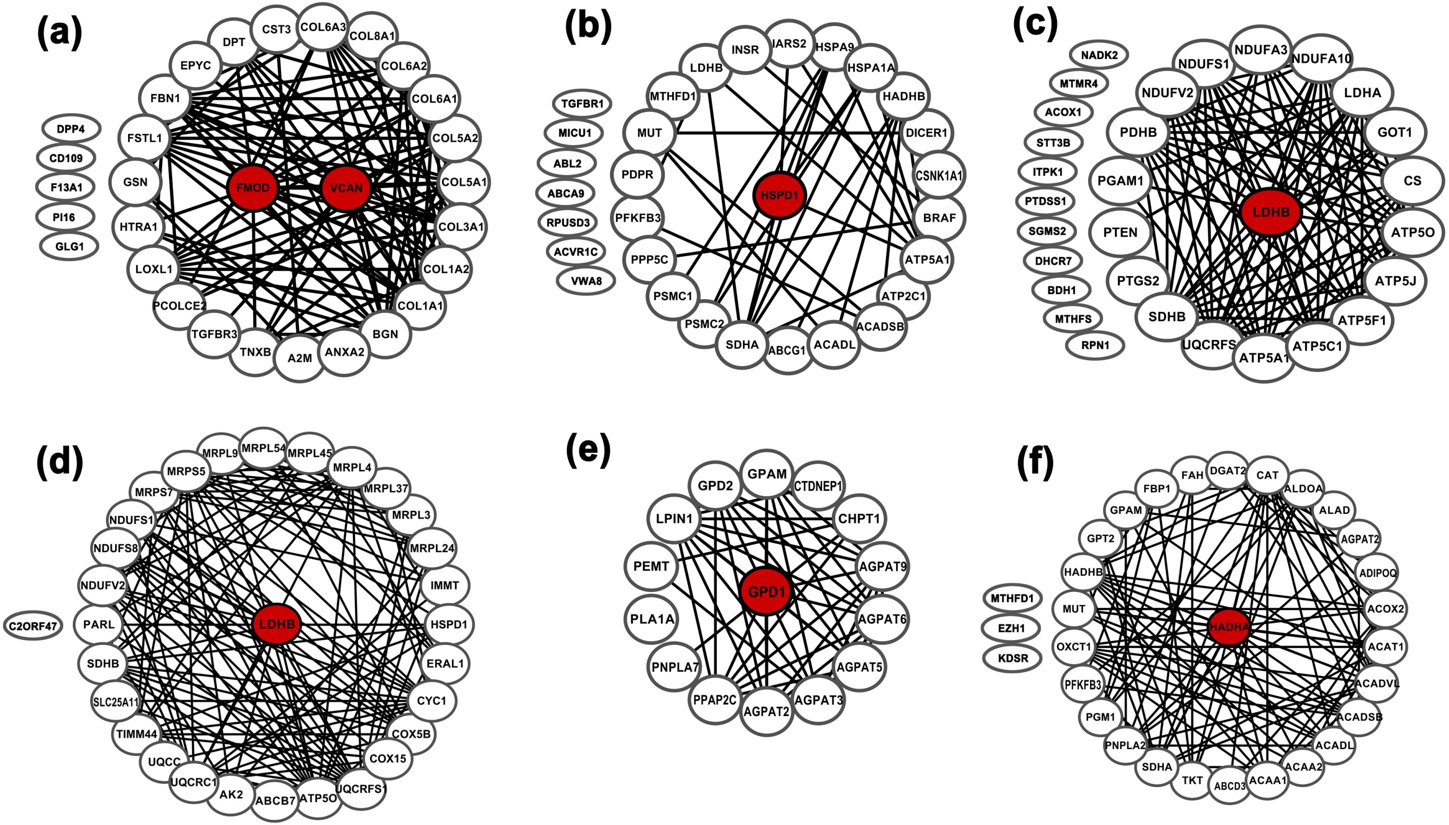
(a)
DPP4
CD109
F13A1
PI16
GLG1
FMOD
VCAN
DPT
CST3
COL6A3
COL8A1
EPYC
COL6A2
FBN1
COL6A1
FSTL1
COL5A2
GSN
COL5A1
HTRA1
COL3A1
LOXL1
COL1A2
PCOLCE2
COL1A1
TGFBR3
BGN
TNXB
A2M
ANXA2
(b)
TGFBR1
MICU1
ABL2
ABCA9
RPUSD3
ACVR1C
VWA8
HSPD1
INSR
IARS2
HSPA9
LDHB
HSPA1A
MTHFD1
HADHB
MUT
DICER1
PDPR
CSNK1A1
PFKFB3
BRAF
PPP5C
ATP5A1
PSMC1
ATP2C1
PSMC2
ACADSB
SDHA
ABCG1
ACADL
(c)
NADK2
MTMR4
ACOX1
STT3B
ITPK1
PTDSS1
SGMS2
DHCR7
BDH1
MTHFS
RPN1
LDHB
NDUFS1
NDUFA3
NDUFA10
NDUFV2
LDHA
PDHB
GOT1
PGAM1
CS
PTEN
ATP5O
PTGS2
ATP5J
SDHB
ATP5F1
UQCRFS
ATP5A1
ATP5C1
(d)
C2ORF47
LDHB
MRPL9
MRPL54
MRPL45
MRPS5
MRPL4
MRPS7
MRPL37
NDUFS1
MRPL3
NDUFS8
MRPL24
NDUFV2
IMMT
PARL
HSPD1
SDHB
ERAL1
SLC25A11
CYC1
TIMM44
COX5B
UQCC
COX15
UQCRC1
AK2
ABCB7
ATP5O
UQCRFS1
(e)
GPD1
GPD2
GPAM
CTDNEP1
LPIN1
CHPT1
PEMT
AGPAT9
PLA1A
AGPAT6
PNPLA7
AGPAT5
PPAP2C
AGPAT2
AGPAT3
(f)
MTHFD1
EZH1
KDSR
HADHA
FAH
DGAT2
CAT
FBP1
ALDOA
GPAM
ALAD
GPT2
AGPAT2
HADHB
ADIPOQ
MUT
ACOX2
OXCT1
ACAT1
PFKFB3
ACADVL
PGM1
ACADSB
PNPLA2
ACADL
SDHA
ACAA2
TKT
ABCD3
ACAA1
